# Screening of Low-Tumorigenic MDCK Cells for Potential Influenza Vaccine Substrates and Transcriptomic Analyses

**DOI:** 10.3390/ijms27093875

**Published:** 2026-04-27

**Authors:** Lei Zhou, Xiaoxi Li, Jianmin Chen, Yuanyuan Liu, Yuming Zhang, Xiaojie Gong, Qingwei Meng, Zhongyu Li

**Affiliations:** 1 School of Chemical Engineering, Dalian University of Technology, Dalian 116600, China; 62245006@mail.dlut.edu.cn; 2 Key Laboratory of Biotechnology and Bioresources Utilization, Ministry of Education, Dalian Minzu University, Dalian 116600, China; 202312040058@stu.dlnu.edu.cn (X.L.); 202312040048@stu.dlnu.edu.cn (J.C.); 202412081007@stu.dlnu.edu.cn (Y.L.); 202412082008@stu.dlnu.edu.cn (Y.Z.); gxjclr@163.com (X.G.)

**Keywords:** MDCK cell, transcriptomic analysis, tumorigenic testing

## Abstract

Since MDCK cells are inherently tumorigenic, their safety in vaccine production has long been a concern; thus, establishing a screening method for low-tumorigenic cells is of great significance for influenza vaccine development. This study successfully obtained a low-tumorigenic MDCK cell line through monoclonal screening and systematically evaluated its potential as a cellular substrate for influenza vaccines using male nude mice (BALB/c nu/nu, 4–7 weeks old) for tumorigenicity assessment. Comprehensive analysis of the biological characteristics of the screened cells—including growth curves and transcriptomic features—showed that the cell line exhibits stable growth and consistent traits. Transcriptomic comparison was performed between two defined biological states: parental MDCK cells (SQ group) and the low-tumorigenic clone MDCK-20B9 (SH group). Transcriptomic analysis revealed good dispersion among samples and an overall consistent gene expression distribution. Differential expression analysis identified a total of 2198 differentially expressed genes, including 902 upregulated and 1296 downregulated genes. GO functional enrichment analysis indicated that these genes are mainly involved in biological processes such as acute-phase response, retinol metabolism, mitotic chromosome condensation, and cell migration; are enriched in cellular components such as kinetochores and the extracellular matrix; and are associated with molecular functions including calcium ion binding and the Wnt signaling pathway. KEGG pathway analysis further revealed that the differentially expressed genes are significantly enriched in key pathways such as cancer pathways, cell cycle, and cell adhesion molecules. The expression trends of five key differentially expressed genes were validated by RT-qPCR. In summary, this study successfully screened a stable and consistent low-tumorigenic MDCK cell line, providing a theoretical basis and practical foundation for its use as a cellular substrate in influenza vaccine development.

## 1. Introduction

Influenza remains a significant global health burden, causing annual epidemics and periodic pandemics that pose substantial threats to both human and animal populations [1]. As one of the most effective interventions for pandemic preparedness, influenza vaccination has demonstrated remarkable efficacy since the landmark 1943 clinical trial conducted among U.S. Army personnel, which reported a 69% reduction in febrile illness (2.2% attack rate in vaccine recipients versus 7.1% in placebo). To date, vaccination remains the most effective public health strategy for preventing viral transmission and reducing disease severity [2,3]. Despite these successes, current vaccine manufacturing faces critical production bottlenecks [4,5]. Although traditional egg-based influenza vaccine production offers advantages such as mature technology, relatively low production costs, and scalable capacity, it still has several limitations [6]. These include susceptibility to microbial contamination, high residual endotoxin levels, poor responsiveness to pandemic outbreaks [7], limited egg supply, a lengthy production cycle of up to 26 weeks, potential egg-derived allergens, and adaptive mutations acquired by the virus during propagation in eggs, which may compromise vaccine efficacy and safety [8,9]. Consequently, this method is gradually failing to meet global influenza vaccine demands.

Safety and efficacy are critical metrics in vaccine research and production, making the use of safe, stable, and effective continuous cell lines (CCLs) for influenza vaccine manufacturing an important development direction [10,11]. Some cell lines, such as BHK-21, CHO, and HeLa, are classified by the World Health Organization (WHO) as tumorigenic cell lines due to their ability to form tumors in immunocompromised animals (e.g., rodents) [12]. The MDCK cell line was established by Madin and Darby in 1958 from the kidney of a female Cocker Spaniel dog [7]. MDCK cells typically grow as adherent epithelial-like cells and are recognized as one of the most suitable cell lines for influenza virus vaccine production due to their high viral infection efficiency, adaptability to serum-free conditions, sensitivity to various influenza virus strains, rapid proliferation, and low tendency for mutation [13]. However, because MDCK cells are tumorigenic, the safety of vaccines produced using them has been widely debated [14].

Several companies, including MedImmune, Novartis, Solvay, and GlaxoSmithKline, have conducted research on using MDCK cells to propagate influenza viruses for vaccine production [15]. Notably, on 1 June 2007, Optaflu, a seasonal influenza vaccine produced by Novartis Vaccines using MDCK cell culture, was approved for use in the European Union by the European Medicines Agency (EMA) [16]. In November 2012, the vaccine was approved by the U.S. Food and Drug Administration (FDA) [U.S. FDA. Vaccines [17]], and renamed Flucelvax. Decades of post-marketing surveillance studies have confirmed its favorable safety profile [18,19]. However, the safety of Flucelvax relies on multiple redundant manufacturing processes—including extensive filtration, chemical inactivation with β-propiolactone, and DNA fragmentation—to reduce the probability of residual viable cells and residual DNA. While these measures effectively mitigate risks, they add considerable complexity and cost to manufacturing. Notably, recent studies have demonstrated that reducing MDCK tumorigenicity through genetic engineering of apoptosis regulators such as Bcl-xL can effectively decrease tumor formation in nude mice from 100% to 10%, highlighting the feasibility and importance of developing enhanced safety substrates. The identification of key tumorigenicity-related genes and microRNAs further supports the rationale for targeted screening strategies to isolate low-tumorigenic variants. The development of explicitly low-tumorigenic or non-tumorigenic MDCK variants would represent a substantial advance by potentially streamlining purification protocols, reducing regulatory scrutiny, and further minimizing theoretical oncogenic risks at the substrate level rather than relying solely on downstream process controls.

From a long-term development perspective, developing low-tumorigenic MDCK cell lines would not only simplify downstream purification processes but also fundamentally reduce the risks associated with influenza vaccines produced from such cells.

Despite considerable efforts to characterize MDCK tumorigenicity, existing studies have yet to establish a fully integrated workflow that seamlessly connects monoclonal screening with rigorous in vivo tumorigenicity validation and molecular marker confirmation. Consequently, critical questions regarding the long-term genetic stability of screened clones and definitive passage limits for manufacturing applications remain unresolved. The present study addresses these gaps by implementing a comprehensive, reproducible pipeline that combines limiting dilution cloning, systematic transcriptomic analysis, and RT-qPCR validation of tumorigenicity-related genes, culminating in a well-defined master cell bank. Therefore, this study offers a practical, scientifically grounded framework for industrial implementation. In this study, we report the establishment of a non-tumorigenic MDCK cell clone through limiting dilution monoclonal screening, coupled with comprehensive characterization including transcriptomic profiling, RT-qPCR validation of tumorigenicity-associated gene expression, and longitudinal assessment of genetic stability across serial passages. This work provides a foundation for developing enhanced biosafety substrates suitable for next-generation influenza vaccine manufacturing.

## 2. Results

### 2.1. Screening of Low-Tumorigenic Cells and Establishment of the Master Cell Bank

Low-tumorigenic cells were screened using a monoclonal method, resulting in the isolation of 19 cell clones and the establishment of a master cell bank. All selected clones exhibited epithelial-like morphology, showing typical fibroblastic characteristics. The viability of cryopreserved cells was not less than 95%, and the freezing density was not less than 1 × 10^6^ cells/mL. The 19 clones refer to the final number of stable, single-cell-derived MDCK subclones successfully expanded to Master Cell Bank stage. Detailed characterization (karyotyping, tumorigenicity assay, transcriptomics) was performed only on MDCK-20B9, as this was our lead candidate. The other 18 clones are maintained in our cryopreserved clone library for future investigation. Low-tumorigenic MDCK cells were isolated by limiting dilution cloning of parental MDCK cells (CCL-34, ATCC, P12 from WCB). Briefly, single-cell suspensions were plated at 1.0 cell/well (nominal) across 480 wells; 247 wells showed cell growth (51.5% plating efficiency), of which 142 were verified single-cell-derived by three-stage microscopic confirmation (Stage I: 4–6 h, single-cell identification; Stage II: 24–48 h, division origin tracking; Stage III: Day 7, colony morphology assessment). Following expansion to T-75 flasks, 19 clones (13.4%) exhibited stable growth without crisis or senescence and were cryopreserved to establish Clone Master Banks (viability ≥ 95%, density ≥ 1 × 10^6^ cells/mL). The remaining 123 verified single-cell-derived clones failed to expand to T-75 due to slow growth, morphological abnormalities, or cryopreservation failure. Preliminary phenotypic screening classified these into three groups: Group A (high-proliferation, serum-independent; 8 clones) resembling parental MDCK; Group B (moderate-proliferation, serum-dependent; 7 clones) requiring 2–5% FBS; and Group C (slow-growth or unstable; 4 clones) excluded from further study. From Group B, Clone 20B9 (MDCK-20B9) was selected for comprehensive characterization based on stable growth across P5–15, consistent epithelial morphology without fibroblast-like transformation, and intermediate expansion ratio (1:10–1:15) suggesting a “normalized” phenotype.

**Figure 1:**
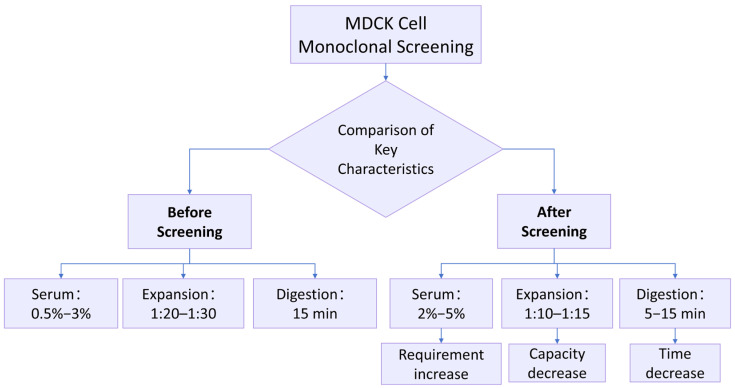
Comparative biological characteristics of Parental MDCK and MDCK-20B9. Flowchart illustrating key phenotypic differences between the parental heterogeneous cell line and the single-cell-derived clone selected for reduced tumorigenic potential. Parental MDCK (high-tumorigenic) demonstrates low serum requirement (0.5–3% FBS), high expansion capacity (1:20–1:30), and extended digestion time (~15 min). MDCK-20B9 (low-tumorigenic) shows increased serum dependency (2–5% FBS), reduced expansion ratio (1:10–1:15), and shortened digestion time (5–10 min), consistent with a less transformed, more contact-inhibited phenotype. Data represent the specific selected clone MDCK-20B9, not multiple individual clones.

Figure 1 presents the comparative biological characteristics of Parental MDCK and the selected clone MDCK-20B9. Following monoclonal screening of 19 candidate clones derived from limiting dilution cloning, Clone 20B9 was selected for comprehensive characterization based on serum dependency, expansion ratio, and epithelial morphology stability. All subsequent detailed analyses—growth curves, karyotype stability, tumorigenicity assessment, and transcriptomic profiling—were performed exclusively on MDCK-20B9 at defined passage levels to establish its suitability as a vaccine substrate.

### 2.2. Biological Characteristics of Parental MDCK and MDCK-20B9

This section compares the biological characteristics of two cell populations: Parental MDCK (SQ group, P14) representing the original high-tumorigenic cell line, and MDCK-20B9 (SH group, P15) representing the single-cell-derived clone selected for reduced tumorigenic potential. Parental MDCK P14 showed 10 of 10 tumorigenic in animal assay and 67.2% *n* = 78 ± 2 karyotype compliance (Section 2.4.1); meanwhile, MDCK-20B9 P15 showed 0 of 10 tumorigenic and 89.5% *n* = 78 ± 2 karyotype compliance, confirming that the growth parameter differences reflect distinct biological states. The biological characteristics of MDCK cells were observed for Parental MDCK and MDCK-20B9. Prior to screening, the cells required a serum supplementation of 0.5% to 3%, achieved an expansion ratio of 1:20 to 1:30, and had a digestion time of approximately 15 min. Following monoclonal screening, the serum requirement increased to a range of 2% to 5%, the expansion ratio decreased to 1:10 to 1:15, and the digestion time shortened to 5–10 min (Figure 1). These phenotypic shifts indicate that MDCK-20B9 exhibits partial reversion toward a normalized, contact-inhibited state with reduced tumorigenic properties. Through the monoclonal screening methodology, the target cell line can be selected for research, development, and production applications.

Observations of the MDCK cell characteristics before and MDCK-20B9 revealed that with increased screening cycles, the required serum concentration gradually increased, the cell expansion ratio progressively decreased, and the digestion time was reduced. This indicates that with more screening cycles, the cells gradually normalized and exhibited low-tumorigenic properties. Through the screening method and multiple screening cycles, the target cell line can be selected for use in research, development, and production, thereby overcoming the “bottleneck” technology issue related to cell seeds.

### 2.3. Growth Curves of MDCK-20B9 During Passaging

**Figure 2:**
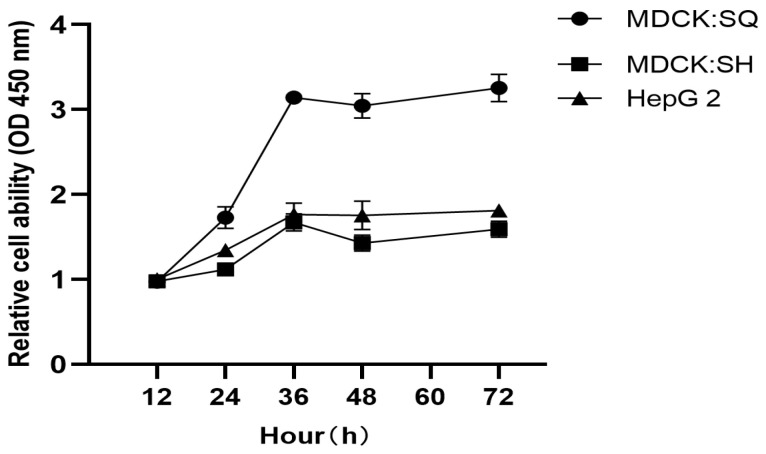
Growth curves of Parental MDCK, MDCK-20B9, and HepG2 cells. Growth kinetics were assessed by CCK-8 assay over 84 h with measurements every 12 h. Parental MDCK (SQ group) shows rapid proliferation with doubling time of 18.4 h. MDCK-20B9 (SH group) demonstrates reduced growth rate with doubling time of 26.7 h, consistent with attenuated transformation status. HepG2 hepatocellular carcinoma cells serve as malignant reference with fastest proliferation. Data points represent mean ± SD of six replicate wells.

Growth curves were generated to compare proliferation kinetics between Parental MDCK (SQ group) and MDCK-20B9 (SH group), using HepG2 hepatocellular carcinoma cells as a reference control. These passage levels were selected to match the tumorigenicity assessment and transcriptomic analysis passages, ensuring data consistency across all characterization experiments. All cultures were maintained at 37 °C with 5% CO_2_. Cell viability was assessed every 12 h using the CCK-8 assay over 84 h (Figure 2). After normalization, all three cell lines exhibited typical sigmoid growth curves. However, MDCK-20B9 (SH) demonstrated reduced growth rate compared to Parental MDCK (SQ), with doubling times of 26.7 h versus 18.4 h respectively, consistent with the less transformed phenotype of the selected clone. HepG2 cells showed the most rapid proliferation as expected for a malignant reference line.

### 2.4. Preliminary Examination of Low-Tumorigenic Cells

#### 2.4.1. Karyotype Analysis

Clone MDCK-20B9 was selected from the 19-candidate panel based on combined performance across three preliminary screening criteria established in Section 2.2. Among the 7 clones in Group B (moderate-proliferation, serum-dependent), MDCK-20B9 exhibited the most favorable profile: serum dependency of 2–3% FBS for optimal growth, indicating partial growth factor dependence suggestive of “normalized” phenotype; expansion ratio of 1:10 to 1:15, representing controlled proliferative capacity intermediate between parental hyperproliferation and senescent underproliferation; and most stable epithelial morphology without fibroblast-like transformation or crisis during expansion to T-75. Additionally, initial karyotype screening at P5 showed 88.9% of cells with *n* = 78 ± 2 chromosomes, the highest compliance among all 19 candidates. These combined characteristics identified MDCK-20B9 as the lead candidate most likely to exhibit stable, reduced tumorigenicity suitable for detailed longitudinal characterization.

Chromosomal karyotype analysis was performed on this low-tumorigenic cell line at different passages (passages 5, 15, 25, 35, 45, 55, and 65). The results showed that for passages 5, 15, and 25, the proportion of cells with *n* = 78 ± 2 was >80%. However, for passages 35, 45, 55, and 65, the karyotype analysis results were ≤80% (detailed results are shown in Table 1). The findings indicate that during cell subculturing, chromosomes remained unchanged up to passage 25, demonstrating good genetic stability. Karyotype analysis suggests that tumorigenicity emerges after passage 35. Therefore, it is recommended that the cell passages used for production and research should not exceed passage 25.

#### 2.4.2. Tumorigenicity Assay in Animals

(1) Pre-Subcloning Tumorigenicity Assessment (Parental MDCK)

A tumorigenicity assay was performed on the cells prior to screening, conducted in accordance with the requirements of the Chinese Pharmacopoeia (2020 Edition, Volume III). The cell line used was Parental MDCK (ATCC CCL-34) at passage 14–16. The positive control was HeLa cells (human cervical carcinoma, ATCC CCL-2) inoculated at 10^7^ cells per mouse to validate assay sensitivity. After a 16-week observation period, typical tumorigenicity was observed. Following the conclusion of the experiment, histopathological sections were prepared from the tumors formed in the animals.

**Figure 3:**
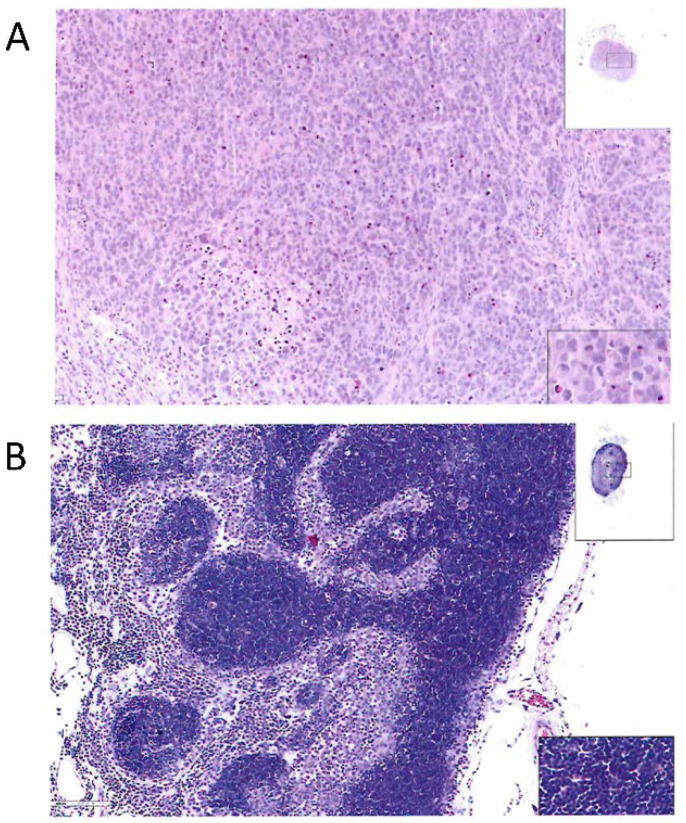
Histopathological examination of tumorigenicity assay in nude mice (HE, 10 × 20). (**A**) Positive control HeLa cells group. (**B**) Parental MDCK cell group. Insets: low-magnification view (**left**) and high-magnification detail of lymphoid follicles (**right**).

The results indicated that in the positive control group (nude mice inoculated with HeLa cells), subcutaneous tumor cells were observed at the inoculation site, showing diffuse growth in clusters and nests. The cell morphology was diverse with marked atypia; the nuclei were large, appearing round, oval, polygonal, etc., with frequent pathological mitotic figures. Focal hemorrhage and necrosis were also observed. Microscopic examination of the liver, kidney, spleen, heart, lungs, mesenteric lymph nodes, and tissues surrounding the inoculation site revealed no significant abnormalities. Microscopic observation of nodules near the inoculation site in nude mice from the Parental MDCK cell group revealed lymph nodes, showing intact cortical and medullary areas. The cortical area displayed clearly defined lymphoid follicles, while the medullary area consisted of medullary cords and sinuses. No significant abnormalities were observed in other organs. The cells under examination exhibited typical tumor characteristics. Histopathological sections are detailed in Figure 3.

(2) MDCK-20B9 Tumorigenicity Assay in Animals

**Figure 4:**
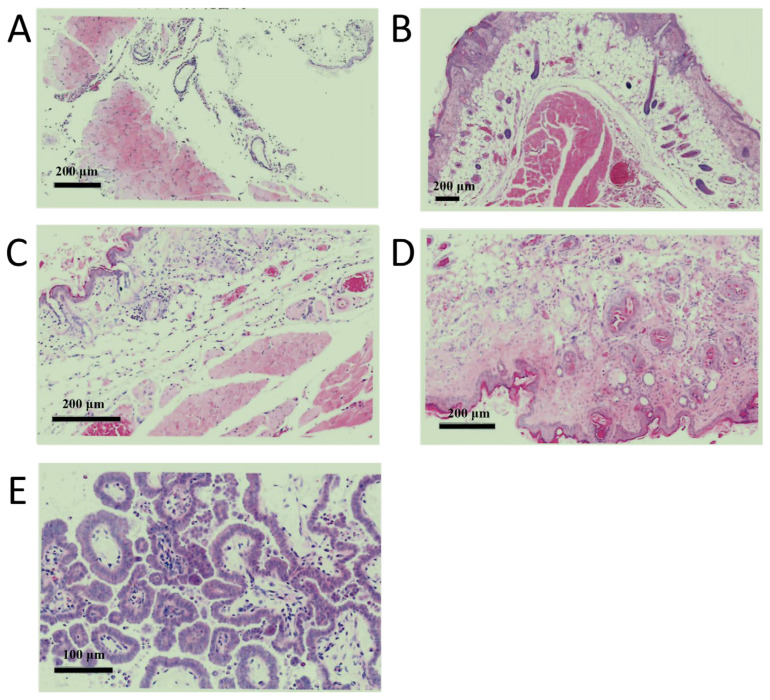
Histopathological examination of inoculation sites in nude mice at different passages of screened MDCK cells. (**A**) Passage 5, Bar = 200 µm, (**B**) Passage 15, Bar = 200 µm, (**C**) Passage 25, Bar = 200 µm, (**D**) Passage 35, Bar = 200 µm and (**E**) Passage 45, Bar = 100 µm.

A tumorigenicity assay was performed in animals using the screened low-tumorigenic cells at different passages (cell passage levels: 5, 15, 25, 35, and 45). For each passage, cells were inoculated at densities of 10^7^ cells to study the effects of different passages and inoculation densities. The results are shown in Figure 4. No abnormal structures were observed at the inoculation sites in animals of Passage 5, Passage 15, Passage 25, and Passage 35. In contrast, abnormal structures were visible at the inoculation sites in mice of Passage 45, where subcutaneous cystic spaces were present, containing abundant pale purple mucoid material. Glandular structures were also observed, with most glandular epithelial cells arranged in a regular monolayer of cuboidal or flattened shape. Locally, areas with multilayered and irregular arrangements were noted, and scattered muciparous cells were seen within the glandular lumina. Under high-power magnification, no significant cellular atypia was observed. Furthermore, microscopic examination of the heart, lungs, liver, spleen, kidneys, and mesenteric lymph nodes revealed no significant abnormalities. Combining the results from the karyotype analysis and the animal tumorigenicity assay, it is recommended that the passage number used for research, development, and production of the screened cells should not exceed 25.

### 2.5. Transcriptomic Assay

The transcriptomic analysis compared six samples in two groups: SQ-1, SQ-2, SQ-3 (biological triplicates of Parental MDCK) and SH-1, SH-2, SH-3 (biological triplicates of MDCK-20B9). SQ-1, SQ-2, and SQ-3 were derived from Parental MDCK (ATCC CCL-34) at passage 14. These cells exhibited high tumorigenicity in the animal assay (10/10 mice, Section 2.4.2), showed rapid growth kinetics (doubling time 18.4 h, Section 2.3), required low serum concentration (0.5–2% FBS, Section 2.2), and demonstrated aneuploid karyotype with 67.2% of cells at *n* = 78 ± 2 (Section 2.4.1). SH-1, SH-2, and SH-3 were derived from MDCK-20B9 at passage 15. This clone was selected from 19 candidates based on moderate serum dependency and stable epithelial morphology (Section 2.1). At P15, MDCK-20B9 exhibited confirmed non-tumorigenicity (0/10 mice, Section 2.4.2), reduced growth kinetics (doubling time 26.7 h, Section 2.3), required moderate serum concentration (2–5% FBS, Section 2.2), and showed near-diploid karyotype with 89.5% of cells at *n* = 78 ± 2 (Section 2.4.1). Eukaryotic reference-based transcriptome (RNA-seq) analysis was performed on six samples, yielding a total of 39.23 Gb of clean data. Each sample provided at least 5.99 Gb of clean data, with the percentage of Q30 bases being 98.25% or higher. The clean reads from each sample were aligned to the designated reference genome, with alignment efficiencies ranging from 97.18% to 97.51%.

Based on the alignment results, analyses for alternative splicing prediction, gene structure optimization, and novel gene discovery were conducted. This process led to the discovery of 3941 novel genes, of which 719 were functionally annotated.

#### 2.5.1. Gene Expression Distribution

**Figure 5:**
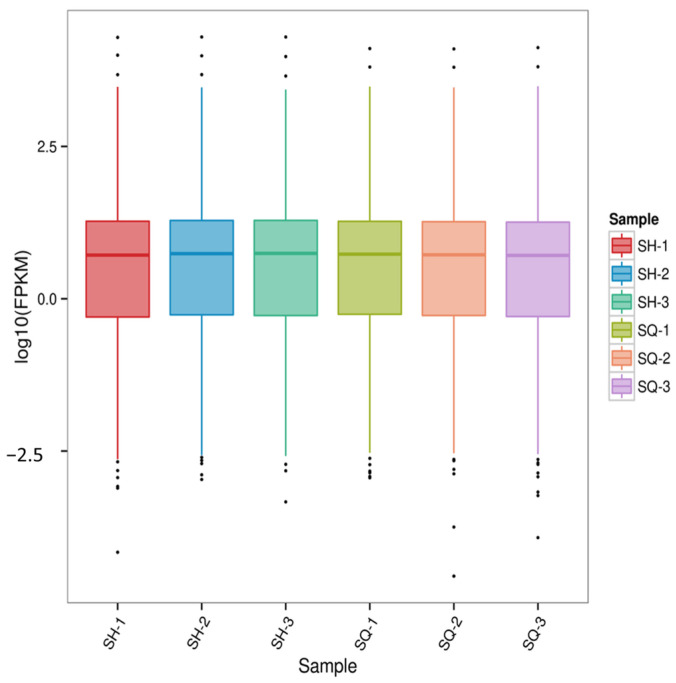
Gene Expression Distribution.

Transcriptomic data enables highly sensitive detection of gene expression. Typically, the expression levels (FPKM values) of protein-coding genes that can be sequenced span six orders of magnitude, from 10^(−2)^ to 10^4^ [20]. Using the Parental MDCK cells (SQ group) as the control for comparison with the screened low-tumorigenic MDCK cells (SH group), the gene expression distribution across all sample groups was examined. Furthermore, an FPKM distribution boxplot (Figure 5) was constructed for the gene expression levels in each sample. This allows not only for assessing the dispersion degree of gene expression level distribution within individual samples but also for an intuitive comparison of the overall gene expression levels across different samples.

Combined analysis of both figures indicates that the expression levels of most genes are concentrated within the range of 0.1 to 10. The distribution of gene expression across samples and the overall gene expression levels are largely consistent.

#### 2.5.2. Evaluation of Replicate Correlation

Studies have shown that the expression of the same gene can vary between individuals due to biological variability [21,22] to identify truly relevant differentially expressed genes, it is necessary to account for and manage expression differences caused by this biological variability [23]. Currently, the most common and effective approach is to include biological replicates in the experimental design.

**Figure 6:**
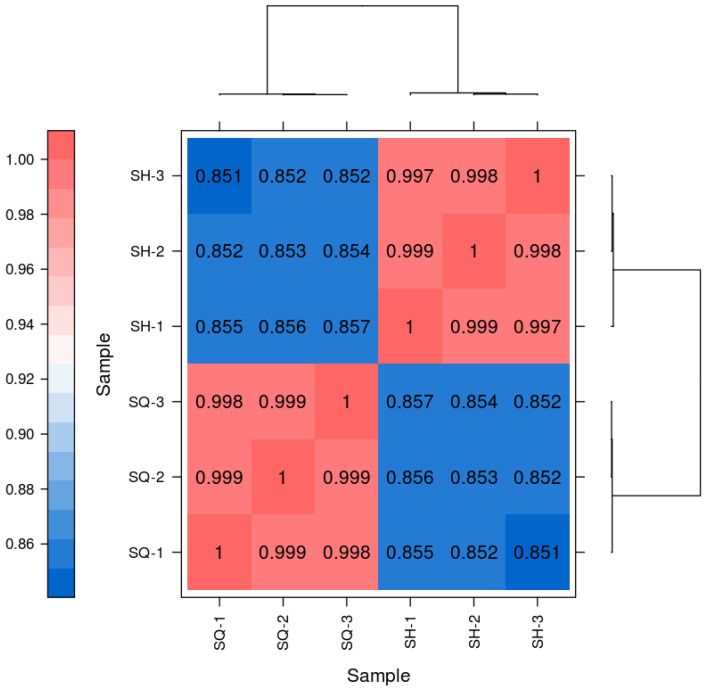
Heatmap of Pairwise Sample Expression Correlation.

The heatmap results, using the square of Pearson’s correlation coefficient (R^2^) as an evaluation metric for replicate correlation [24], indicate that an R^2^ value closer to 1 signifies a stronger correlation between two replicate samples. The R^2^ values obtained in this experiment were greater than 0.8. As shown in Figure 6, SQ-1, SQ-2, and SQ-3 exhibited high correlation with each other, and similarly, SH-1, SH-2, and SH-3 showed high mutual correlation. In contrast, the correlation between the SQ group and the SH group was very low. These results meet the requirements for analysis, demonstrating strong gene expression level correlation among the biological replicate samples.

#### 2.5.3. Differential Gene Expression Analysis

Transcriptomic comparison was performed between two defined biological states: parental MDCK cells (SQ group) and the low-tumorigenic clone MDCK-20B9 (SH group). Differentially expressed genes were identified based on gene count values across samples using differential analysis software. For comparison groups with biological replicates, differential analysis was performed using DESeq2 [25]; for groups without biological replicates, edgeR was employed [26]. This direct comparison identifies molecular alterations associated with the reduced tumorigenic phenotype achieved through monoclonal screening. Differential expression analysis using DESeq2 identified 2198 differentially ex-pressed genes (DEGs) with stringent criteria: absolute log2 fold change ≥ 1 and false discovery rate < 0.05, comprising 902 upregulated and 1296 downregulated genes in SH relative to SQ.

Transcriptomic comparison was performed between two defined biological states: parental MDCK cells (SQ group, high-tumorigenic, passage 14, *n* = 3 biological replicates) and the low-tumorigenic clone MDCK-20B9 (SH group, passage 15, *n* = 3 biological replicates). This direct comparison identifies molecular alterations associated with the reduced tumorigenic phenotype achieved through monoclonal screening. Differential expression analysis using DESeq2 identified 2198 differentially expressed genes (DEGs) with stringent criteria of absolute log2 fold change ≥ 1 and false discovery rate < 0.05, comprising 902 upregulated and 1296 downregulated genes in SH relative to SQ.

**Figure 7:**
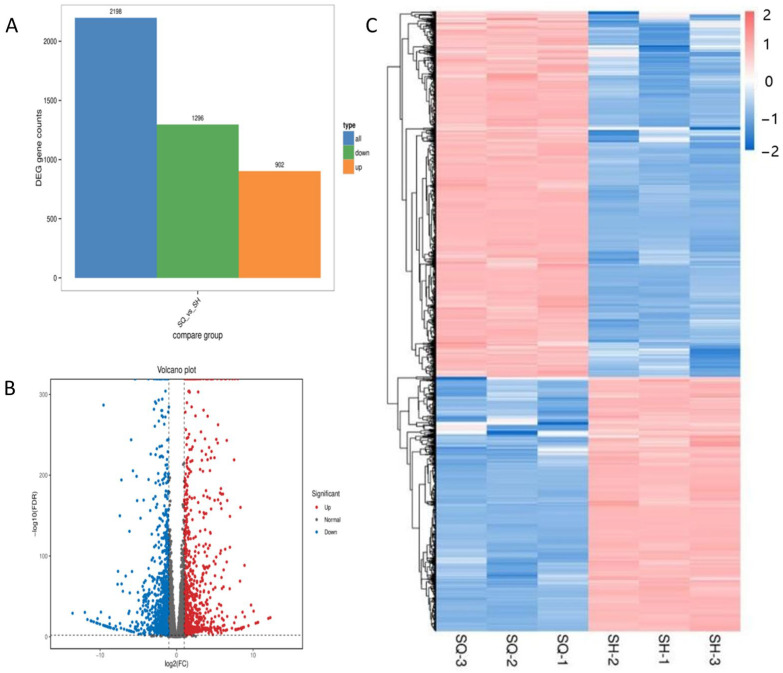
Differential gene expression analysis comparing parental MDCK (SQ, high-tumorigenic) and screened MDCK-20B9 (SH, low-tumorigenic) cells. (**A**) Volcano plot showing statistical significance (−log10 FDR) versus magnitude of expression change (log2 fold change); red points indicate 902 genes significantly upregulated in SH, blue points indicate 1296 genes significantly downregulated, with threshold lines at log2FC = ±1 and FDR = 0.05. (**B**) Hierarchical clustering heatmap of the top 200 differentially expressed genes after Z-score normalization of FPKM values, demonstrating distinct transcriptional profiles between SQ replicates (SQ-1, SQ-2, SQ-3) and SH replicates (SH-1, SH-2, SH-3). (**C**) MA plot showing the relationship between mean expression level (log2 average FPKM) and fold change (log2FC), with DEGs highlighted in red.

Figure 7 presents three complementary visualization strategies to characterize these DEGs. Panel A (volcano plot) displays the complete transcriptomic landscape with statistical significance (−log10 FDR) on the y-axis and expression change (log2 fold change) on the x-axis; vertical threshold lines at log2FC = ±1 and horizontal threshold at FDR = 0.05 demarcate significant DEGs, with red indicating upregulation and blue indicating downregulation in SH. Panel B (hierarchical clustering heatmap) shows expression patterns of the top 200 DEGs across all six samples after Z-score normalization of FPKM values, demonstrating clear segregation of SQ and SH replicates into distinct transcriptional clusters with high within-group correlation. Panel C (MA plot) illustrates the relationship between mean expression level (log2 average FPKM) and fold change (log2FC), confirming that differential expression is distributed across the full dynamic range without bias toward highly or lowly expressed genes.

The differential expression patterns correlate with the observed phenotypic divergence between parental and screened cells. Upregulated genes in SH include pro-apoptotic regulators (CYCS, TNFRSF10A) and cell cycle checkpoints, consistent with the reduced proliferation rate and confirmed non-tumorigenicity in nude mice; downregulated genes include AKT and other proliferative signaling components, aligning with increased serum dependency (2–5% versus 0.5–3% FBS) and decreased expansion ratio (1:10–15 versus 1:20–30) documented in biological characterization. This integrated analysis establishes that monoclonal screening induced substantial transcriptional reprogramming toward a less transformed, more contact-inhibited cellular state.

#### 2.5.4. Enrichment Analysis of Differentially Expressed Genes

The COG (Clusters of Orthologous Groups) database can be used to classify gene products based on orthologous relationships. The distribution of genes across different functional categories reflects the metabolic or physiological priorities under specific conditions or stages. To further investigate the core functional roles of the differentially expressed genes (DEGs), functional annotation and classification were performed using the COG database.

**Figure 8:**
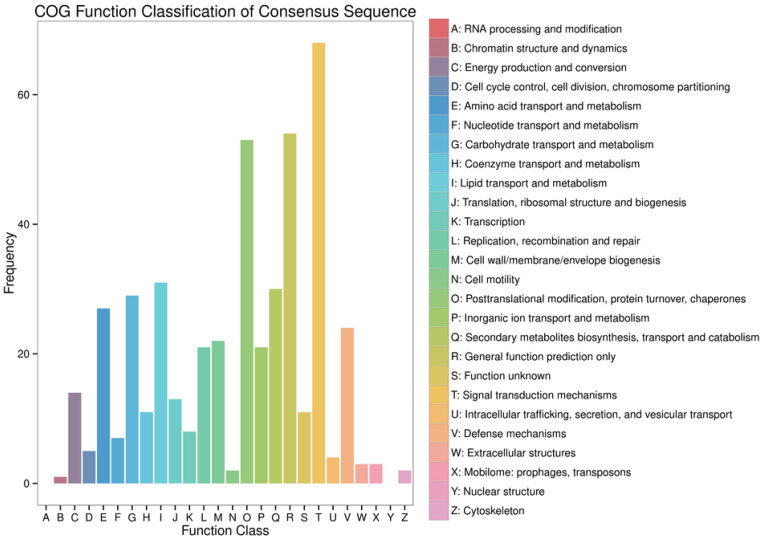
Statistical Chart of COG Annotation and Classification for Differentially Expressed Genes.

As shown in Figure 8, among the 26 annotated functional categories, the DEGs were primarily enriched in the following: Signal transduction mechanisms (T, 68 genes); General function prediction only (R, 54 genes); Posttranslational modification, protein turnover, chaperones (O, 53 genes); Lipid transport and metabolism (I, 31 genes); Secondary metabolites biosynthesis, transport, and catabolism (Q, 30 genes); and Carbohydrate transport and metabolism (G, 29 genes). Notably, a significant enrichment of DEGs was observed in the signal transduction mechanisms category, suggesting potential alterations in the regulation of relevant signaling pathways in the screened low-tumorigenic MDCK cell line.

To elucidate the specific biological functions of the DEGs, Gene Ontology (GO) functional classification and annotation were conducted from three aspects: biological process, cellular component, and molecular function. The significance of functional pathways is generally determined by the q-value, with smaller q-values indicating greater significance.

**Figure 9:**
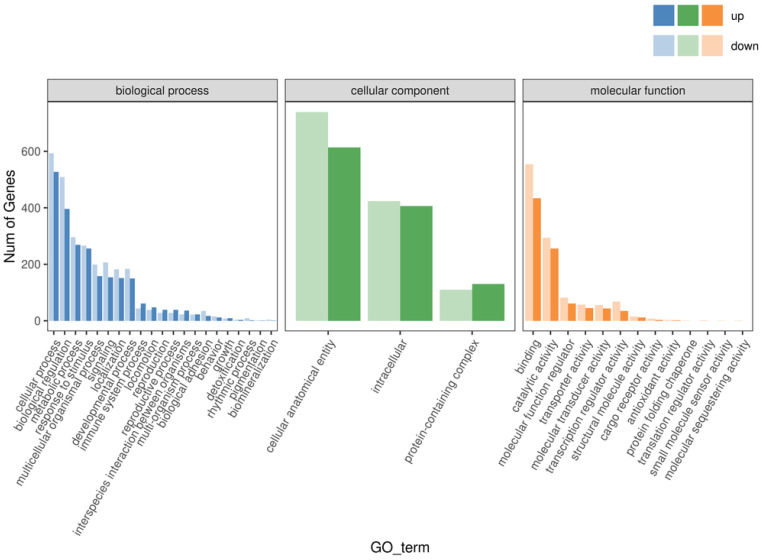
Statistical Chart of GO Annotation and Classification for Differentially Expressed Genes.

The results are shown in Figure 9. Among the annotated DEGs in the “Biological Process” category, the majority were concentrated in cellular processes (1120 genes), biological regulation (905 genes), metabolic processes (565 genes), and response to stimulus (522 genes). In the “Cellular Component” category, a higher number of DEGs were enriched in cellular anatomical entity (1353 genes), intracellular component (830 genes), and protein-containing complex (240 genes). In the “Molecular Function” category, DEGs were mainly involved in binding (988 genes), catalytic activity (550 genes), molecular function regulator (143 genes), transcription regulator activity (103 genes), and transporter activity (102 genes).

GO enrichment analysis based on the transcriptomic data revealed a coordinated regulatory pattern involving both up-regulated and down-regulated DEGs. In the screened low-tumorigenic MDCK cell line, changes were observed in genes highly associated with tumorigenicity, such as those involved in cellular processes and metabolic processes.

**Figure 10:**
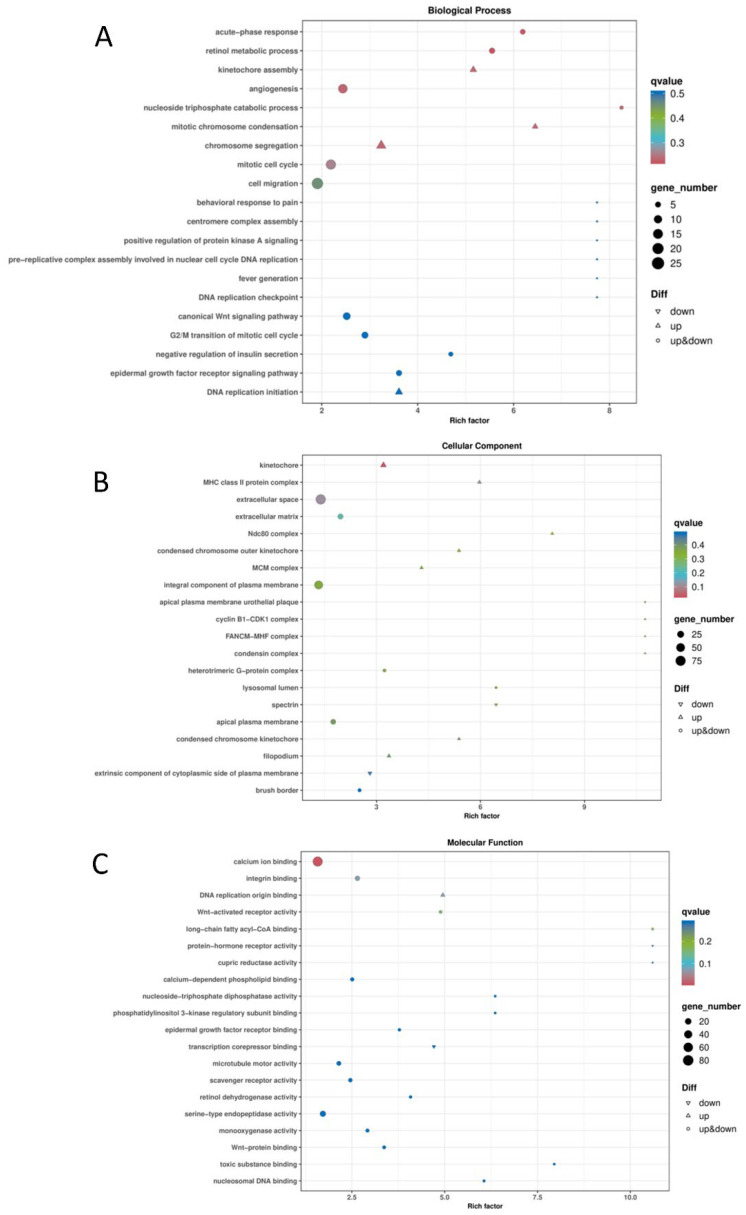
Bubble Chart of GO Enrichment for Differentially Expressed Genes. (**A**) Biological Process (BP); (**B**) Cellular Component (CC); (**C**) Molecular Function (MF).

**Figure 11:**
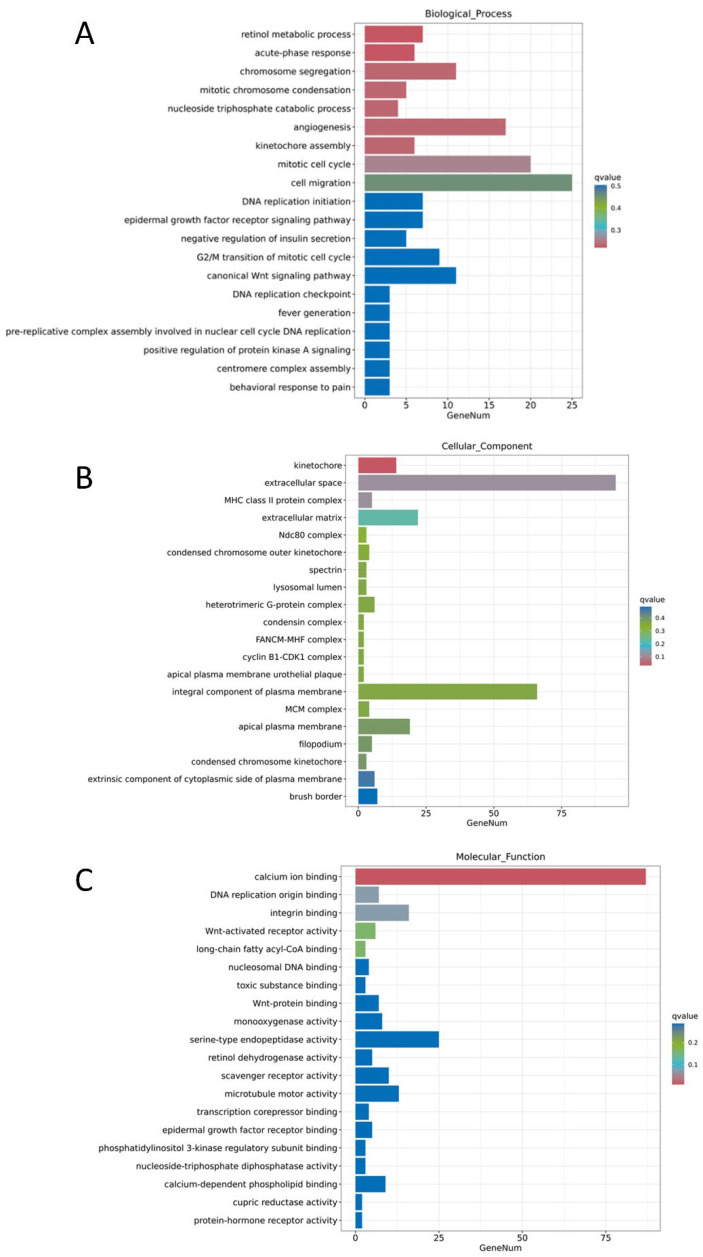
Bar chart of GO enrichment for differentially expressed genes. (**A**) Biological Process (BP); (**B**) Cellular Component (CC); (**C**) Molecular Function (MF).

**Figure 12:**
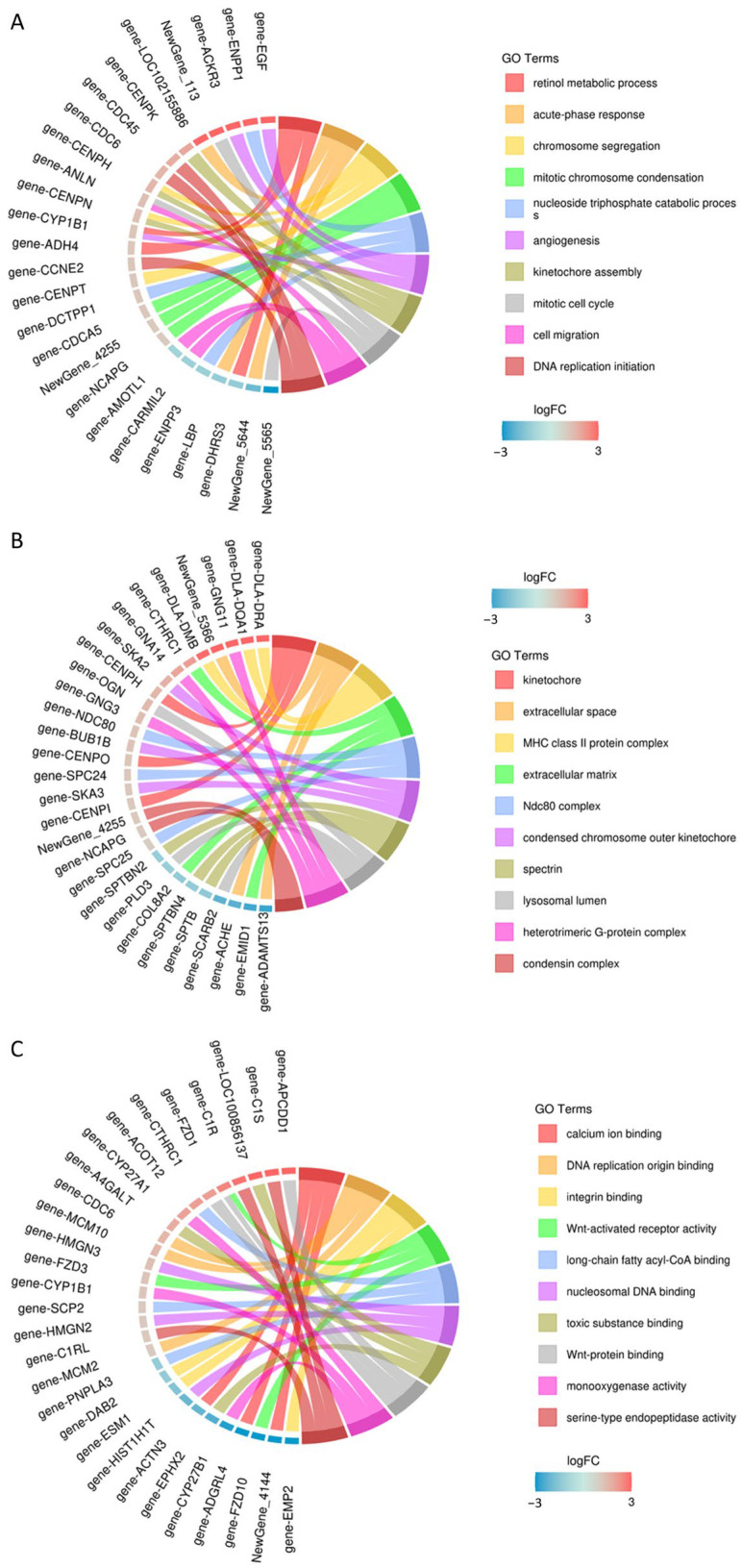
Enrichment chord plot of GO terms for differentially expressed genes. (**A**) Biological Process (BP); (**B**) Cellular Component (CC); (**C**) Molecular Function (MF).

To understand the functional distribution of differentially expressed genes in the screened MDCK cells, the Cluster Profiler tool was employed. Using the hypergeometric test method, GO enrichment analysis was performed on the differentially expressed gene sets from each group, examining the three major categories: Biological Process (BP), Cellular Component (CC), and Molecular Function (MF). Visualization of the enrichment results was achieved using various plots, including bubble charts (Figure 10), bar charts (Figure 11), and enrichment circle plots (Figure 12).

GO analysis of the differentially expressed genes revealed the following associations: In terms of Biological Process (BP), the genes were primarily involved in the acute-phase response, retinol metabolic process, nucleoside triphosphate catabolic process, mitotic chromosome condensation, and cell migration. For Cellular Component (CC), enrichment was mainly observed in the kinetochore, extracellular space, extracellular matrix, Ndc80 complex, and condensin complex. Regarding Molecular Function (MF), the genes were chiefly associated with calcium ion binding, Wnt-activated receptor activity, serine-type endopeptidase activity, and Wnt-protein binding (Figure 10).

Figure 11 displays the proportion of related genes among all differentially expressed genes. The top 10 enriched GO terms were selected to create enrichment circle plots (Figure 11). For BP, the main terms involved were retinol metabolic process, acute-phase response, chromosome segregation, mitotic chromosome condensation, nucleoside triphosphate catabolic process, angiogenesis, kinetochore assembly, mitotic cell cycle, cell migration, and DNA replication initiation. For CC, the primary terms included kinetochore, extracellular space, extracellular matrix, MHC class II protein complex, Ndc80 complex, condensed chromosome outer kinetochore, condensin complex, heterotrimeric G-protein complex, lysosomal lumen, and spectrin. For MF, the main terms encompassed binding to calcium ions, integrins, Wnt proteins, toxic substances, DNA replication origins, long-chain fatty acyl-CoA, and nucleosome DNA, as well as activities such as Wnt-activated receptor activity, serine-type endopeptidase activity, and monooxygenase activity (Figure 12).

#### 2.5.5. KEGG Classification and Annotation

The up-regulated and down-regulated differentially expressed genes (DEGs) were separately mapped to the KEGG database. The KEGG annotation results for the DEGs were categorized according to the pathway types in KEGG to further interpret gene functions, which include the metabolism of carbohydrates, nucleotides, and amino acids, as well as the biodegradation of organic compounds.

**Figure 13:**
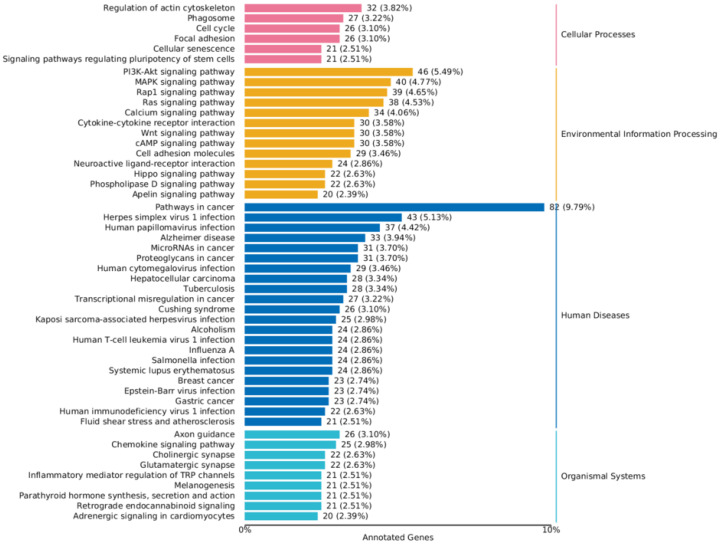
KEGG classification chart of differentially expressed genes.

The results revealed that in the screened MDCK cells, a total of 50 pathways were significantly enriched (*p* < 0.05). Among these, 6 pathways were related to Cellular Processes (e.g., Cell cycle, Autophagy), 13 pathways were related to Environmental Information Processing (e.g., Signal transduction, Membrane transport), 22 pathways were related to Human Diseases, and 9 pathways were related to Organismal Systems (e.g., Digestive system, Nervous system signal transmission) (Figure 13).

Using pathways from the KEGG database as units, a hypergeometric test was applied to identify pathways that were significantly enriched in the differentially expressed genes compared to the whole genomic background. Pathway significance enrichment can determine the primary biochemical metabolic and signal transduction pathways in which the genes are involved. The enrichment results were visualized using bubble charts, bar charts, and network diagrams with ClusterProfiler.

**Figure 14:**
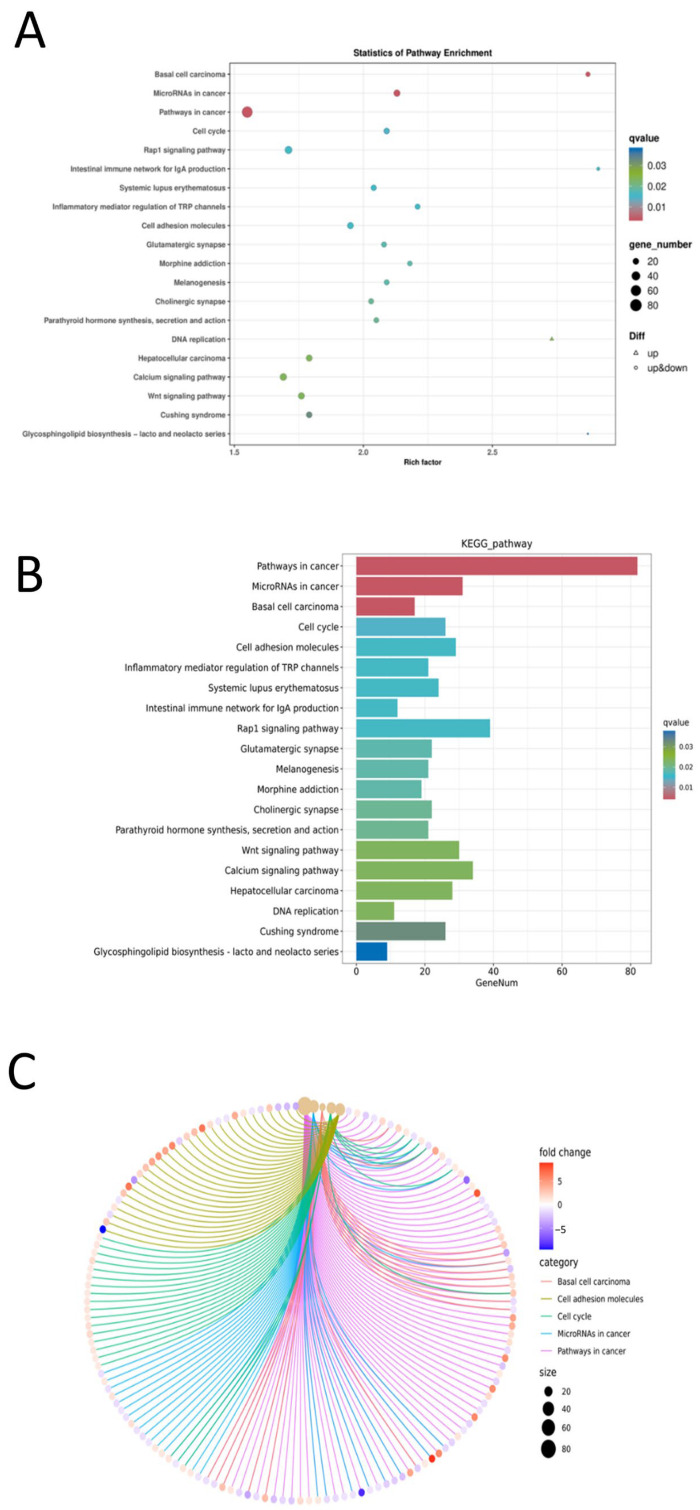
Results of KEGG enrichment analysis. (**A**) Bubble chart of KEGG enrichment for differentially expressed genes; (**B**) Bar chart of KEGG enrichment for differentially expressed genes; (**C**) Network diagram of KEGG enrichment for differentially expressed genes.

**Figure 15:**
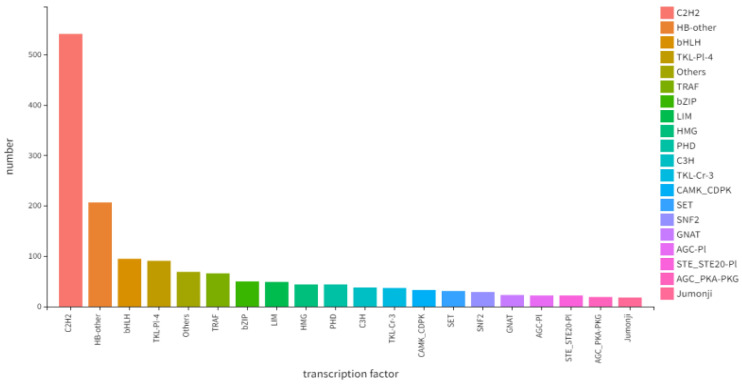
Prediction of transcription factors.

Focusing on the top 20 pathways with the smallest q-values, a KEGG pathway enrichment analysis of the differentially expressed genes was conducted to identify the main biochemical metabolic and signal transduction pathways involved. The results revealed that the five most significantly enriched pathways (by q-value) were: Pathways in cancer; MicroRNAs in cancer; Basal cell carcinoma; Cell cycle; and Cell adhesion molecules (Figure 14). Prediction of transcription factors indicated that C2H2 was the most abundant, with the numbers of other factors decreasing sequentially thereafter (Figure 15).

### 2.6. Detection of Target Gene Expression by RT-qPCR

Based on the transcriptomic analysis results, five key differentially expressed genes associated with tumorigenicity were selected for further validation. Among them, four genes were found to be up-regulated: cytochrome c (CYCS), B-cell lymphoma 2 (BCL2), insulin receptor (INSR), and tumor necrosis factor receptor superfamily member 10A (TNFRSF10A). In contrast, protein kinase B (AKT) was identified as a down-regulated gene. These genes are known to play critical roles in apoptosis, metabolic signaling, and cell survival pathways.

**Figure 16:**
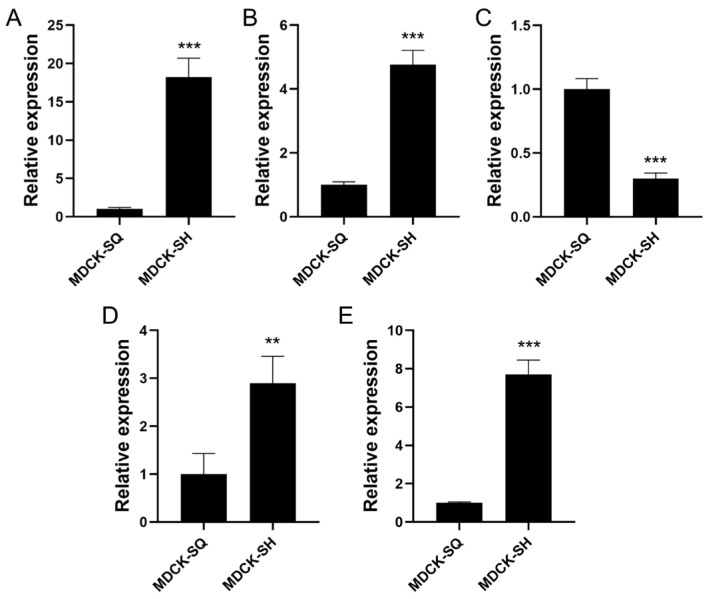
Validation results of differentially expressed genes in MDCK cells. (**A**) CYCS, (**B**) BCL2, (**C**) AKT, (**D**) INSR, (**E**) TNFRSF10A. (Values are the means ± SEM, *n* = 3. ** *p* < 0.01, *** *p* < 0.001, SH vs SQ).

To validate the RNA-seq data, reverse transcription-quantitative PCR (RT-qPCR) was performed to examine the expression levels of these five candidate genes. The RT-qPCR results exhibited expression trends consistent with those observed in the transcriptomic analysis (Figure 16), thereby confirming the reliability of the sequencing data.

## 3. Discussion

The inherent tumorigenicity of MDCK cells has long been a subject of regulatory and scientific debate regarding their suitability as substrates for human vaccine production. While the approved MDCK-derived vaccine Optaflu/Flucelvax has demonstrated favorable post-marketing safety profiles, the development of explicitly non-tumorigenic cell variants represents a proactive strategy to further mitigate theoretical risks, streamline downstream purification requirements, and potentially accelerate regulatory approval pathways for future cell-based influenza vaccines. This study establishes a robust, integrated methodology for isolating and characterizing non-tumorigenic MDCK cells, addressing a critical unmet need in the field.

We successfully employed limiting dilution cloning to isolate a non-tumorigenic MDCK subclone and established a qualified master cell bank. Comprehensive biological characterization revealed that the screened cells exhibited stable growth kinetics with a characteristic S-shaped curve, albeit with reduced proliferation rates compared to parental tumorigenic cells—consistent with a less transformed phenotype. Notably, the screened cells demonstrated altered culture requirements, including increased serum dependence (2–5% versus 0.5–3%), reduced expansion capacity (1:10–1:15 versus 1:20–1:30), and shortened trypsinization times (5–10 versus ~15 min). These phenotypic shifts suggest a partial reversion toward a more differentiated, contact-inhibited state that correlates with attenuated tumorigenic potential [4].

The transcriptomic analysis provided robust, reproducible data supporting the biological distinction between parental tumorigenic cells (SQ) and screened low-tumorigenic cells (SH) populations. High inter-replicate correlation (R^2^ > 0.85) within groups and marked divergence between groups (R^2^ < 0.86) confirmed the reliability of our differential expression analysis. The identification of 2198 DEGs—comprising 902 upregulated and 1296 downregulated genes—revealed extensive rewiring of cellular processes associated with transformation. GO enrichment analysis highlighted significant alterations in acute-phase response, retinol metabolism, and mitotic chromosome condensation (BP); kinetochore and extracellular matrix components (CC); and calcium ion binding together with Wnt signaling activities (MF). These findings align with established mechanisms of tumorigenesis, including dysregulated cell cycle control, altered metabolic programming, and disrupted cell–cell/cell–matrix interactions.

KEGG pathway analysis further corroborated these observations, with prominent enrichment in cancer-associated pathways, cell cycle regulation, and cell adhesion molecules. The differential expression of key tumorigenicity markers—including upregulation of pro-apoptotic CYCS, anti-apoptotic BCL2, and TNFRSF10A, alongside downregulation of proliferative driver AKT—was independently validated by RT-qPCR. These expression patterns collectively suggest a shift toward reduced malignant potential in the screened clone, though the precise functional consequences of these alterations warrant further mechanistic investigation.

Critical to industrial translation, our longitudinal stability assessment established P25 as the maximum safe passage limit for manufacturing applications. Karyotype analysis revealed stable chromosome numbers (*n* = 78 ± 2 in >80% of cells) through P25, with progressive chromosomal drift and emergence of tumorigenicity in nude mice occurring beyond this threshold. This finding has immediate practical implications: it defines a clear, evidence-based boundary for cell culture operations and underscores the necessity of rigorous passage monitoring in GMP environments. The observed genetic instability at higher passages likely reflects clonal selection pressures and accumulated epigenetic alterations during extended culture, phenomena well-documented in continuous cell lines.

It is essential to contextualize this passage limitation within standard industrial practice. All continuous cell lines employed in vaccine manufacturing operate under validated passage restrictions. Vero cells, despite being classified as non-tumorigenic, are typically restricted to P140–P150 for regulatory compliance, and diploid MRC-5 cells have finite lifespans of approximately 50–60 population doublings. The value of our methodology lies not in achieving infinite genetic stability—which is biologically unattainable for any continuous cell line—but in establishing rigorous, evidence-based boundaries that enable reproducible, scalable manufacturing. The P25 limit for MDCK-20B9 represents a conservative safety margin: at P25, cells maintain both karyotype stability (>80% *n* = 78 ± 2) and confirmed non-tumorigenicity (0/10 mice), providing assurance before the P35 threshold where instability emerges.

Industrial scalability is achievable through standard cell banking paradigms. Implementation would proceed as follows: (1) establish a fully qualified Master Cell Bank (MCB) at P15–P20 with comprehensive characterization including karyotype, tumorigenicity, and transcriptomic profiling; (2) derive Working Cell Banks (WCB) with confirmed P25 maximum passage limit and routine quality monitoring; (3) implement karyotype analysis as an in-process control for extended production campaigns. Given MDCK-20B9 doubling times of 24–30 h, the 25-passage window from WCB thawing to production harvest supports approximately 50–60 population doublings—sufficient for multi-thousand-liter bioreactor campaigns typical of influenza vaccine manufacturing. This framework directly parallels established regulatory practice for MDCK, Vero, and other continuous cell substrates, where finite passage limits are accepted as manageable operational constraints rather than fundamental barriers to industrial application.

Several limitations of this study should be acknowledged. While chromosome number stability correlates with phenotypic behavior in our longitudinal study, oncogenic rearrangements may occur without net chromosome number change. First, while the nude mouse tumorigenicity assay remains the regulatory gold standard, it represents an immunocompromised model that may not fully predict behavior in immunocompetent hosts; orthogonal assays such as soft agar colony formation or chick embryo chorioallantoic membrane assays could provide complementary data. Second, the transcriptomic analysis, while comprehensive, represents a single timepoint snapshot; dynamic gene expression profiling across the culture lifespan might reveal additional predictive markers of instability. Third, functional validation of the DEGs through gene editing or overexpression studies was beyond our current scope but would strengthen causal inferences regarding tumorigenicity mechanisms. Finally, viral productivity and antigenic fidelity—critical parameters for vaccine manufacturing—remain to be evaluated in the non-tumorigenic substrate, though prior literature suggests MDCK cells generally maintain robust influenza virus support regardless of transformation status. Future work should employ G-banding or whole-genome sequencing to comprehensively characterize genomic stability. Future studies will evaluate influenza virus growth kinetics and antigen yield in MDCK-20B9 to confirm that the reduced tumorigenic phenotype does not compromise viral productivity, a critical parameter for vaccine manufacturing that was beyond the scope of the current safety-focused characterization.

## 4. Materials and Methods

### 4.1. Cell Culture

The MDCK cells (CCL-34) were obtained from the American Type Culture Collection (ATCC). The MDCK cells (CCL-34) were obtained from the American Type Culture Collection (ATCC). Upon receipt, cells were expanded and cryopreserved to establish a Master Cell Bank (MCB) and a Working Cell Bank (WCB). All experiments were initiated from the WCB at passages 11–13. Cells were maintained in Dulbecco’s Modified Eagle Medium (DMEM, Gibco, Waltham, MA, USA) supplemented with 10% (*v*/*v*) fetal bovine serum (FBS, Lonza, Guangzhou, China), 2 mM L-glutamine (Gibco), 100 U/mL penicillin and 100 μg/mL streptomycin (Gibco), and 1 mM sodium pyruvate (Gibco). Cells were cultured at 37 °C in a humidified atmosphere of 5% CO_2_, with medium replacement every 48 h. At 80–90% confluence, cells were washed twice with phosphate-buffered saline and incubated with 0.25% trypsin-EDTA (Gibco) at 37 °C for 3–5 min. Trypsinization was terminated by addition of complete medium at a 1:3 volume ratio. Cells were pelleted by centrifugation at 3000 rpm for 5 min, resuspended in fresh medium. Subcultivation was performed at a split ratio of 1:5 to 1:10 every 3–4 days.

### 4.2. Major Reagents and Equipment

Trypsin was sourced from Gibco; MDCK cell culture medium was supplied by Aopumai (Shanghai, China) and Bailing (Lanzhou, China); and fetal bovine serum was obtained from Lonza (Guangzhou, China).

### 4.3. Limiting Dilution Cloning and Single-Cell Verification

Single-cell cloning was performed using the classical limiting dilution method. Briefly, parental MDCK cells (P12, from WCB) grown to 80–90% confluence were washed with PBS, digested with 0.25% trypsin-EDTA at 37 °C for 5–10 min, and resuspended in complete medium. Following centrifugation at 200× *g* for 5 min, cells were counted and serially diluted to a nominal concentration of 5 cells/mL. Using a multichannel pipette, 200 μL aliquots were distributed into 96-well flat-bottom plates (480 wells total across 5 plates), yielding a nominal density of 1.0 cell/well.

Clonal origin was verified through a rigorous three-stage microscopic protocol: (i) 4–6 h post-plating, phase-contrast microscopy (10×) identified wells containing exactly one viable, attached cell (142/247 attachment-positive wells met criterion); (ii) 24–48 h later, re-examination confirmed single-point origin of cell division with adjacent, radially symmetric daughter cells (97/142 confirmed); and (iii) Day 7 assessment retained only compact, circular colonies with uniform cobblestone morphology (84/97 confirmed as single-cell-derived). Verified clones were expanded through sequential passage (96-well→24-well→6-well→T-25→T-75), with 19 clones exhibiting stable growth characteristics suitable for establishment of Clone Master Banks at ~30 population doublings.

### 4.4. Comparative Biological Characteristics of Parental MDCK and MDCK-20B9

The biological characteristics of the cells were observed both before and after the screening process. The serum dependency assay was adapted from Freshney’s Culture of Animal Cells: A Manual of Basic Technique and Specialized Applications, which measures cell proliferation across a dilution series of serum concentrations as an indicator of transformation status. The expansion ratio determination follows the subcultivation recommendations for MDCK cells (CCL-34) provided by ATCC, which we quantified as the maximum sustainable dilution factor. The growth curve analysis using the CCK-8 assay kit (Dojindo, Kumamoto, Japan, CK04) was performed according to the manufacturer’s protocol based on the water-soluble tetrazolium salt WST-8, a method widely applied for cell proliferation assessment. These observations included aspects such as the serum supplementation ratio during cell passaging, the cell expansion ratio, and the cell digestion time.

### 4.5. Cell Growth and Proliferation Assays

The screened cells underwent continuous passaging. Parental tumorigenic cells, screened low-tumorigenic cells, and HepG2 hepatocellular carcinoma cells were cultured separately. All cultures were maintained in a 37 °C incubator with 5% CO_2_. Cell viability was assessed every 12 h using the CCK-8 assay, with measurements taken continuously over a period of 3 days.

Serum Dependency Assay. Cells were seeded at 5 × 10^3^ cells/well in 96-well plates with DMEM containing 0%, 0.5%, 1%, 2%, 5%, or 10% FBS (triplicate wells per condition), incubated for 72 h at 37 °C with 5% CO_2_, and viability measured by CCK-8 assay (Dojindo, CK04; 10 μL reagent added, incubated 2 h, absorbance at 450 nm with reference 650 nm). Serum requirement was defined as the minimum %FBS yielding >80% of maximum observed absorbance (10% FBS), serving as an indicator of growth factor autonomy associated with transformed phenotype.

Expansion Ratio and Growth Curve Analysis. For expansion ratio determination, cells at 90% confluence were trypsinized, counted, and seeded at 1:5, 1:10, 1:20, and 1:30 dilutions from confluent T-75 flasks; time to next 90% confluence was recorded, with expansion ratio reported as the maximum dilution achieving confluence within 72 h at >95% viability. For growth curves, cells were seeded at 1 × 10^4^ cells/well in 96-well plates (6 wells per time point), and CCK-8 assay performed every 12 h for 84 h. Absorbance (450–650 nm) was converted to relative cell number using a standard curve (R^2^ > 0.99); doubling time was calculated from the exponential phase (24–48 h) using DT = t × ln(2)/ln(Nt/N_0_), and growth curves fitted to a modified Richards equation for sigmoid analysis.

### 4.6. Low Tumorigenicity Analysis

(1) Cell Chromosome Karyotype Analysis (at different passage numbers: P5, P15, P25, P35, P45, P55, and P65)

With reference to relevant literature [27], it is recommended to initially screen monoclonal cells using karyotype analysis. The method is as follows: when cells reach 70–80% confluence, colchicine is added, and incubation continues in a 37 ± 1 °C CO_2_ incubator for 2–8 h. Cells are then digested, subjected to hypotonic treatment with saturated potassium chloride, fixed, stained, and finally photographed and counted.

(2) Animal-Based Tumorigenicity Assay

A tumorigenicity assay in animals should be conducted on parental MDCK. MDCK-20B9, cells at different passage numbers (P5, P15, P25, P35, P45) are also subjected to the animal-based tumorigenicity assay to evaluate the stability of the cells MDCK-20B9. Parental MDCK was 10^7^ cells per mouse (5 × 10^7^ cells/mL × 0.2 mL injection volume).

The tumorigenicity test is performed according to the Chinese Pharmacopoeia (2020 Edition, Part III) method for tumorigenicity testing. Nude mice (BALB/c nu/nu, male, 4–7 weeks old) were obtained from Liaoning Changsheng Co., Ltd. (Shengyang, China) and maintained in a specific pathogen-free (SPF) facility at Dalian Minzu University. Animals were housed in individually ventilated cages with controlled environmental conditions: temperature 22 ± 2 °C, relative humidity 50 ± 10%, and a 12 h light/dark cycle. Animals were acclimatized for 7 days prior to experimentation. Nude mice aged 4–7 weeks are selected, with at least 10 mice per group. Animals were randomly allocated to experimental groups using computer-generated random numbers (Microsoft Excel RAND function). For the test cell group, each nude mouse is injected subcutaneously with 0.2 mL of the test cells (i.e., each mouse receives 10^7^ viable cells). For the positive control group (using Hela cells), each mouse is injected with 0.2 mL of positive control cells, containing 10^7^ viable cells. For subcutaneous inoculation, cells should be injected into the dorsal region of the nude mice. For the negative control group (using MRC-5 cells), each mouse is injected with 0.2 mL of negative control cells, containing 5 × 10^7^ viable cells. Animals are observed for at least 16 weeks (at least 4 months), monitoring the injection sites and palpating all animals for any nodule formation at the injection sites. Results are then evaluated.

Animal Care and Monitoring. Inoculation procedures were performed under brief isoflurane anesthesia (2–3% in oxygen) to minimize procedural distress. Humane endpoints were established a priori and included: (1) tumor diameter exceeding 1.5 cm or interfering with normal movement; (2) body weight loss greater than 20% of initial weight; (3) signs of ulceration, infection, or necrosis at the tumor site; (4) severe lethargy or inability to access food and water. Animals were monitored daily for signs of distress and weekly for palpable nodules at injection sites. No unexpected adverse events were observed during the study.

### 4.7. Transcriptomic Analysis

Transcriptomic correlation analysis was then performed on the two groups. The cells were cultured for 48 h. Total RNA was extracted using the RNAprep Pure Cell/Bacteria Kit (Tiangen, Beijing, China) and sent to Huada Gene Company (Shenzhen, China) for cDNA library establishment and whole-transcriptome sequencing. Total RNA was detected using RNA Nano 6000 Assay Kit (Agilent, Santa Clara, CA, USA) and Agilent Bioanalyzer 2100 system. The raw transcriptome data was preprocessed to obtain clean sequences, and short sequence alignment software was used for alignment. The resulting alignment file was used for transcript construction, and the expression of each gene was calculated using the Cufflinks program for subsequent analysis. Differentially expressed genes were identified using the DESeq2 method with filter conditions of |log2 (fold change)| > 2 and adjusted *p* < 0.05. Cluster and volcano plots were generated using the R package “ggplot2” (version 3.4.0), and R software (version 4.2.1) was used for cluster analysis and GO enrichment analysis. This included analyses of gene expression distribution, assessment of replicate correlation, differential gene expression analysis, enrichment analysis of differentially expressed genes, and KEGG classification annotation.

### 4.8. RT-qPCR for Detecting Target Gene Expression Levels

RT-qPCR was employed to measure the expression levels of core differentially expressed genes identified through bioinformatics analysis. This was done to investigate whether the expression of these genes aligned with the bioinformatic predictions. Total RNA was extracted using TRIzol reagent (Invitrogen, Waltham, MA, USA, Cat#15596026) followed by DNase I treatment (RQ1 RNase-free DNase, Promega, Tiangen Biotech, Beijing, China, Cat#M6101) to eliminate genomic DNA contamination. RNA integrity was verified by agarose gel electrophoresis (28S:18S ratio > 1.8) and quantified by NanoDrop spectrophotometry (A260/A280 1.9–2.1). Reverse transcription was performed using High-Capacity cDNA Reverse Transcription Kit (Applied Biosystems, Tiangen Biotech, Beijing, China, Cat#4368814) with random primers in 20 μL reactions according to manufacturer protocol. PowerUp SYBR Green Master Mix (Thermo Fisher, Waltham, MA, USA, Cat#A25742) in 10 μL reactions, 200 nM final primer concentration, 50 °C for 2 min (UDG activation), 95 °C for 2 min (polymerase activation), followed by 40 cycles of 95 °C for 15 s and 60 °C for 1 min (annealing/extension). Melting curve analysis was performed (95 °C for 15 s, 60 °C for 1 min, ramp to 95 °C at 0.15 °C/s). All reactions were performed in technical triplicate on a QuantStudio 3 system (Applied Biosystems).

### 4.9. Statistics and Analysis

All experiments were performed in triplicate or more. Statistical analyses were conducted using one-way ANOVA, with significance levels defined as: * *p* < 0.05, ** *p* < 0.01, *** *p* < 0.001, **** *p* < 0.0001.

## 5. Conclusions

In conclusion, this study presents a reproducible, end-to-end workflow for generating and qualifying non-tumorigenic MDCK cell substrates, from monoclonal isolation through molecular characterization to stability-demarcated master cell banking. By integrating stringent in vivo validation with transcriptomic biomarker profiling, we provide both a practical cellular substrate for influenza vaccine development and a template methodology applicable to cell line qualification more broadly. The establishment of a defined P25 passage limit offers immediate guidance for bioprocess development, while the molecular signatures identified herein may inform future high-throughput screening platforms. These advances collectively support the continued evolution of MDCK-based manufacturing toward enhanced biosafety profiles without compromising production efficiency.

## Figures and Tables

**Table 1:** Longitudinal Karyotype Analysis of MDCK-20B9 at Serial Passages.

Cell Passage	Screened Well ID	Proportion of *n* = 78 (%)	Proportion of *n* = 78 ± 2 (%)	Meets Standard
Passage 5	20B9	34.5	81.8	Yes
Passage 15	20B9	29.8	89.5	Yes
Passage 25	20B9	24.0	81.5	Yes
Passage 35	20B9	40.0	80.0	No
Passage 45	20B9	31.3	64.7	No
Passage 55	20B9	38.0	80.0	No
Passage 65	20B9	24.6	76.3	No

## Data Availability

The study protocol, including research question, key design features, and analysis plan, was prepared prior to experimentation but not registered in a public repository. All other data supporting the findings of this study are available from the corresponding authors upon reasonable request. Individual animal data and histopathological images are available upon request.
